# Treatment of Labor Delayed by Rigidity of the Cervix

**Published:** 1890-11

**Authors:** 


TREATMENT OF LABOR DELAYED BY RIGIDITY OF THE CERVIX.—Dr. W. S. Playfair, in a discussion by the Section of Obstetrics of the British Medical Association, said that in 1874 he had directed attention to the value of chloral in labor. Since that time he has used it constantly with the best results and without trouble from rigidity of the cervix. Under the use of this agent the pains become longer, steadier and more efficient, and the patient falls into a somnolent condition, dozing quietly between the pains, which are not lessened or annulled, as is often the case when chloroform is used. More important than all, the wild stage of excitement which is so frequent in cases of rigid cervix is calmed, to the relief not only of the patient, but of the practitioner. It is not necessary to administer large does; fifteen grains, repeated in twenty minutes, by either the mouth or the rectum, produce an effect that usually lasts for several hours. Possibly a third dose may be required, but never more. Another good effect of this drug is that when the expulsive stage is reached, the patient being already in a state of partial anæsthesia, much smaller quantities of an anæsthetic are required than would otherwise be the case.

Dr. Playfair also referred to the use of quinine, which, he said, acts rather as a general stimulant and promoter of vital energy than as an oxytocic, but in cases of labor with feeble, ineffective pains in the first stage, one or two doses of fifteen grains often have a marked effect in strengthening the pains.

Dr. Robert Bell said that the employment of strychnine in small doses for two or three weeks before labor has a wonderful effect in promoting uterine action. In labor delayed by rigidity of the cervix, although he has had good results from the use of chloral, he now uses tincture of gelsemium in from five to ten drop doses, repeated at intervals of twenty minutes. —*British Medical Journal*, September 27, 1890.

